# Beyond Strength: Toughening Bioactive Ceramics for Functional Bone Repair

**DOI:** 10.34133/bmef.0316

**Published:** 2026-10-14

**Authors:** Xiao Han, Song Chen, Bin Li

**Affiliations:** Medical 3D Printing Center, Orthopedic Institute, Department of Orthopedic Surgery, The First Affiliated Hospital, School of Basic Medical Sciences, Interdisciplinary Innovation Center for Nanomedicine, MOE Key Laboratory of Geriatric Diseases and Immunology, Suzhou Medical College, Soochow University, Suzhou, Jiangsu 215000, China.

As bone-defect repair shifts from structural replacement toward functional regeneration, the design priorities of orthopedic biomaterials are undergoing a fundamental transition [1,2]. Owing to their osteoconductivity, bioactivity, and biodegradability, bioceramics have become one of the most established material platforms for bone repair [3,4]. However, simultaneously achieving high biological activity, mechanical reliability, and long-term stability remains a major challenge.

This transition is driven by the expanding clinical scope of bone-repair bioceramics. Their applications are expanding from structural filling in non-load-bearing defect to mechanically demanding scenarios, such as vertebral augmentation, reconstruction of weight-bearing long bones, and repair after pelvic tumor resection [5]. Meanwhile, the field is increasingly focused on achieving functional bone regeneration in challenging pathological microenvironments, including osteoporosis, chronic inflammation, infection-associated bone defects, and age-related degenerative disorders. Under these conditions, conventional bioceramics are required not only to provide static mechanical support but also to withstand dynamic physiological loading [6]. However, the intrinsic brittleness of hydroxyapatite, β-tricalcium phosphate, bioactive glass, and related ceramics makes them susceptible to microcrack propagation, interfacial failure, and fatigue fracture under cyclic loading and complex stress fields. These failure modes will damage the integrity of the implant, reduce its long-term durability, and ultimately limit its application in load-bearing bone repair [7,8]. Therefore, the future bioceramic design should not only optimize a single component but also integrate multifaceted strategies such as structural enhancement, interface engineering, and dynamic mechanical adaptation.

Natural bone rarely relies on a single strengthening mechanism, instead dissipating mechanical energy across multiple length scales through its hierarchical structure [9]. As a highly sophisticated organic-inorganic composite, carbonate-substituted hydroxyapatite nanocrystals provide rigidity and strength, and Type I collagen provides deformability and toughness. These two phases have a hierarchical structure from nanometer to macro, where multiple enhancement mechanisms work together to suppress crack germination and expansion [10,11]. In contrast, most current bioceramics mainly imitate the inorganic components of bones, and the calcium phosphate phase is commonly used. To improve mechanical strength, high-temperature sintering (more than 800 °C) is often used for densification. Although this process can improve the compressive strength, it also increases the brittleness. Once the local stress exceeds the critical value, the material is prone to catastrophic fractures [12,13]. Many researchers use physical mixing, impregnation, or surface coating to combine ceramic particles with polymers, which include collagen, gelatin, and polylactic acid [14,15]. However, these approaches often lead to poor interface bonding of heterogeneous composite materials. Without strong chemical bonds and effective topological interlocking, the organic-inorganic interface may become a weak region, facilitating crack initiation and propagation rather than suppressing them. Therefore, the next generation of bone repair bioceramics should not only imitate the mineral composition of bone but also accurately reproduce its hierarchical structure, interfacial organization, and synergistic deformation mechanisms (Fig. 1).

**Fig. 1.**
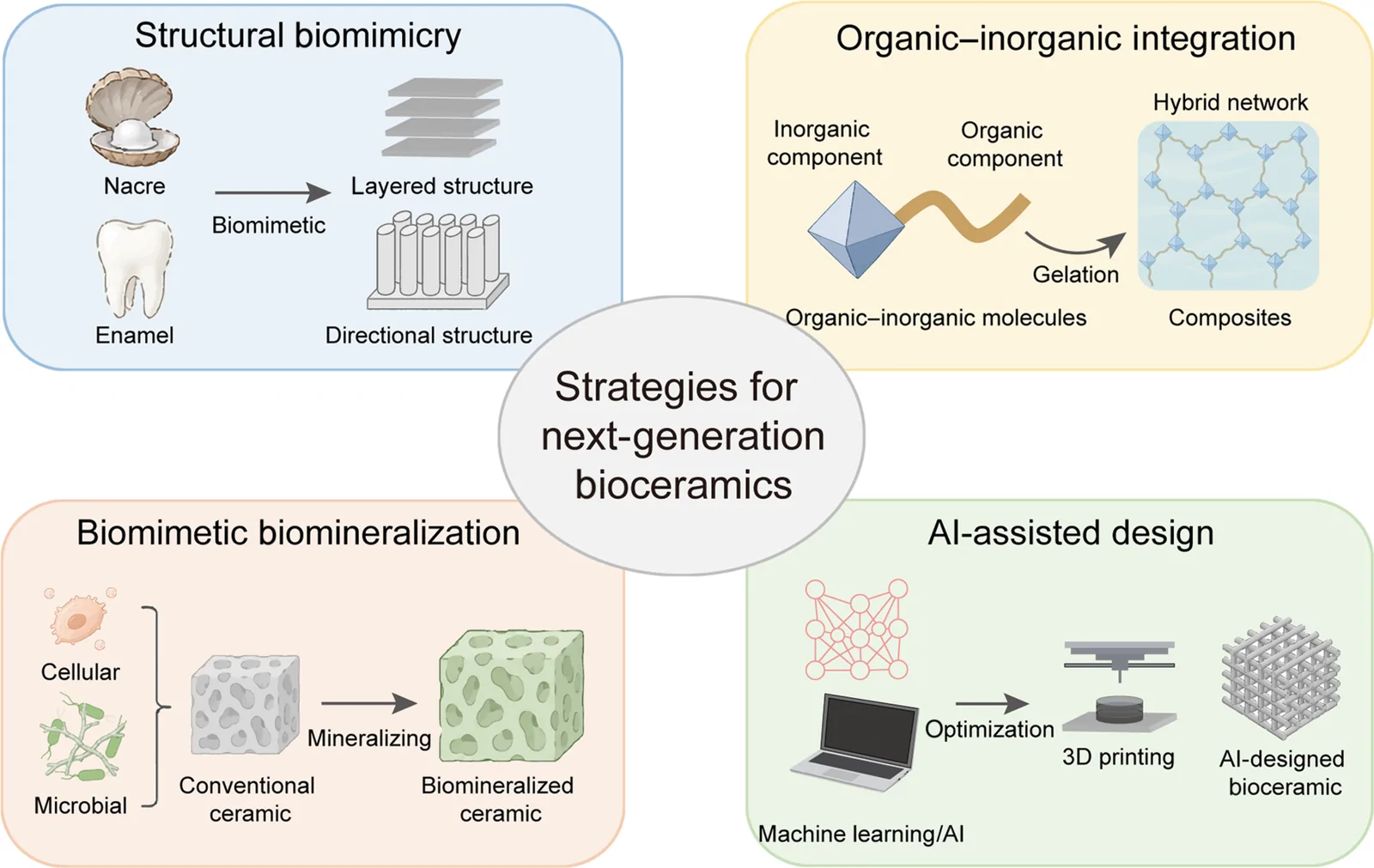
Schematic illustration of four strategies for the preparation of novel bioceramics: Structural Biomimicry [16,19], Organic-Inorganic Integration [21], Biomimetic Mineralization [25,26], and AI-assisted Design [28,29].

## Structural Biomimicry: Beyond Mineral Replication Toward Tissue-Architecture Reconstruction

Inspired by the hierarchical architecture of natural biominerals, including bone, enamel, and nacre, various strategies, such as ice templating, directional freeze casting, and magnetic assembly, have been developed to fabricate bioceramics with ordered lamellar, wavy, and helical microstructures [16]. Nacre-inspired hydroxyapatite/alumina composite ceramics exhibited a 3- to 5-fold increase in fracture toughness through crack deflection at weak interfaces, fiber bridging, and mineral overgrowth, achieving toughness values approaching those of cortical bone [17]. Inspired by the continuous organic-inorganic mineral architecture of the native fibrocartilage interface, a biomineralized collagen scaffold was developed to reproduce aligned mineral organization, thereby directing endogenous fibrocartilage differentiation and enabling functional interfacial regeneration [18]. In another study, hydroxyapatite nanowires coated with an amorphous intergranular phase were combined with polyvinyl alcohol and assembled through directional freezing to construct an enamel-like hierarchical composite spanning the atomic to macroscopic scale [19]. These studies indicate that structural biomimicry is moving beyond simple mineral replication toward the reconstruction of tissue-level architecture. By precisely controlling structural alignment, continuity, and interfacial organization across multiple length scales, bioceramics can simultaneously achieve enhanced mechanical resilience and improved biological performance, providing a robust structural foundation for functional bone regeneration. However, merely relying on the hierarchical structural reinforcement may not ensure long-term damage tolerance, as strong chemical interactions and effective interfacial integration between the organic and inorganic phases are crucial for stable load transfer and crack resistance.

## Homogeneous Organic-Inorganic Integration: From Simple Composites to Genuine Hybrid Materials

Conventional bioceramic composites often show insufficient mechanical properties due to the fundamental mismatch between the rigid inorganic phase and the flexible organic matrix [20]. Ceramics mixed with polymers could increase toughness, but the interface bonding of physical mixing is weak. The load is difficult to transmit, and the interphase zone becomes a weak link in the generation and development of cracks. To meet this challenge, a continuous covalent-ionic network was constructed by integrating covalent and ionic interactions in a single organic-inorganic hybrid molecule. This molecular integrated structure combines ceramics hardness, rubber-like elasticity, and plastics-like processability. Such a combination gives rise to a brand-new material called “elastic ceramic plastic” [21]. In another study, inorganic hydroxyapatite and polyvinyl alcohol were used to simulate the rigid crystalline regions and flexible amorphous regions found in natural mineralized tissues. By achieving a close combination of the two phases, the mineralized structure achieves high tensile strength and high toughness, while also inhibiting the expansion of transverse cracks [22]. These studies indicate that homogeneous organic-inorganic integration at the molecular and nanoscale levels could overcome interfacial mechanical mismatch, which is promising for the design of tough, damage-tolerant bioceramics.

## Biomimetic Mineralization: Learning from Cellular and Microbial Processes

Bone mineralization is a highly regulated biological process rather than a simple inorganic precipitation. Osteoblasts organize a collagen-rich extracellular matrix and release matrix vesicles that locally enrich Ca^2+^ and phosphate ions, providing sites for apatite nucleation and directly contributing to extracellular mineral formation [23]. For bioceramic design, this suggests that mimicking the spatial regulation of mineral nucleation may be just as important as reproducing the mineral composition of bone [24].

Microorganisms provide another route for biologically regulated mineral formation. Urease-producing bacteria have been used to generate uniform CaCO_3_ micro/nanolayers on 2- and 3-dimensional (3D) α-CaSiO_3_ bioceramics, which slowed material degradation and enhanced bone-marrow-mesenchymal-stem-cell adhesion, osteogenic differentiation, and bone formation [25]. More recently, fungal mycelia were incorporated into 3D-printed β-tricalcium phosphate scaffolds, where the growing network contributed to a hierarchical porous structure while releasing bioactive nutrients that supported osteogenesis [26]. Such mineralized structures may also strengthen local interfaces and hinder microcrack propagation, thereby improving mechanical durability. Compared with conventional mineral deposition, these living systems allow mineral formation and structural organization to proceed under much milder conditions.

Although cellular and microbial mineralization rely on different biological mechanisms, both highlight the importance of controlling where and how minerals form. Cell-mediated mineralization provides clues for controlling local ion enrichment and nucleation, whereas microbial systems offer a practical strategy of introducing biologically regulated mineral deposition and interface modification into ceramic materials. These ideas may help guide bioceramics toward more controlled mineral growth and interfacial organization, with potential benefits for bioactivity, degradation, and mechanical durability.

## Artificial-Intelligence-Assisted Design: From Empirical Optimization to Data-Driven Materials Discovery

Biomimetic bioceramic design has advanced rapidly in recent years. However, establishing rational composition-structure-property relationships remains challenging because of the high dimensionality and strong coupling among material parameters. Rather than relying on conventional trial-and-error optimization, artificial intelligence (AI) and machine-learning approaches are enabling a transition toward data-driven materials discovery and inverse design. For example, Pareto set learning was combined with active learning to efficiently explore high-dimensional composition-property relationships in polymer composites, enabling mechanical trade-offs to be resolved through stress-adaptive interfaces [27]. AI can also be used to link material composition, microstructure, and processing parameters with mechanical properties such as strength, toughness, and fatigue resistance, enabling more efficient mechanical optimization of bioceramics. For example, an XGBoost model was used to establish a nonlinear mapping between structural parameters and osteoinductive performance in bioceramics, suggesting that bone-regeneration capacity is primarily governed by porosity and specific surface area, whereas pore geometry plays a comparatively minor role [28]. Further integration with generative design could enable the inverse generation of 3D architectures tailored to specific toughening mechanisms, such as crack deflection and bridging. Coupled with high-throughput simulation and 3D-printing validation, AI may help bridge biomimetic principles with the rapid manufacturing of clinically relevant ceramic implants [29].

Strength remains a fundamental requirement for load-bearing bone-repair materials. However, future material design should consider toughness as an essential complement to strength, particularly in mechanically demanding applications. Toughness should therefore evolve from a mechanical descriptor into a comprehensive design philosophy encompassing hierarchical architecture, interfacial chemistry, dynamic mineralization, and intelligent materials discovery. Combining hierarchical structures, organic-inorganic integration, microbial mineralization, and AI-assisted design may enable multiscale and multifunctional toughening of future bioceramics. Achieving this goal will require continued advances in multiscale structural design, low-temperature hybrid manufacturing, and intelligent materials discovery, as well as deeper integration across materials science, mechanics, biology, and clinical medicine. Future advances in bone-repair bioceramics will depend not only on stronger materials but also on materials that are tougher, more resilient, and better adapted to the biological and mechanical demands of the host environment.
